# Improved Water, Sanitation and Utilization of Maternal and Child Health Services in South Asia—An Analysis of Demographic Health Surveys

**DOI:** 10.3390/ijerph18147667

**Published:** 2021-07-19

**Authors:** Negar Omidakhsh, Ondine S. von Ehrenstein

**Affiliations:** Fielding School of Public Health, University of California, Los Angeles, CA 90095, USA; nomid@ph.ucla.edu

**Keywords:** maternal child health, water, sanitation, health services

## Abstract

Globally, many millions of people still lack access to safe drinking water and sanitation facilities. Here, we examined associations between household availability of improved drinking water and sanitation, respectively, and use of maternal and child health (MCH) services in South Asian countries. Demographic and Health Survey population-based data from Bangladesh, Nepal, India, and Pakistan were used, restricted to women with a child aged 0–36 months (*n* = 145,262). Types of households’ water source and sanitation facilities were categorized based on the World Health Organization and UNICEF’s definitions of “improved” and “unimproved”. We applied logistic regressions to estimate odds ratios (OR) and 95% confidence intervals (CI) for improved water and sanitation, respectively, and reported antenatal care visits, having a skilled attendant at birth, and infant vaccination coverage, stratified by maternal education. Among lower educated women, access to improved water source was associated with greater ORs for presence of a skilled attendant at delivery and their children having up-to-date immunizations (OR: 1.29; 95% CI: 1.17, 1.42). Among lower and higher educated women, improved sanitation (vs. unimproved) was associated with greater ORs for having had adequate antenatal care visits (OR: 1.74; 95% CI: 1.62, 1.88; OR: 1.71; 95% CI: 1.62, 1.80), and similarly for having had a skilled attendant at birth, and children with up-to-date immunizations. Approaches addressing water/sanitation and MCH services across sectors could be a suggested public health strategy.

## 1. Introduction

Globally, it is estimated that over 700 million people lack access to improved drinking water, and that three billion do not have access to improved sanitation, putting particularly women and children in low- and low middle-income countries at increased risk for adverse health outcomes [1]. As it has been recently highlighted, “water serves as an (often) unacknowledged but essential connecting factor for attaining the different Sustainable Development Goals ” [2]; in particular, for attaining Sustainable Development Goal (SDG) 3 targeting the reduction of maternal, neonatal, and child mortality [3].

Infant mortality, a key indicator of population level maternal and child health, has decreased from over 8 million in 1990 to an estimated 4 million annually with a striking gap in rates between countries in northwestern Europe (~1.5–2 deaths/1000 live births (lb)) and South Asian countries, including India (40 deaths/1000 lb) or Nepal (32/1000 lb) [4,5,6,7]. In South Asia, many millions of women and children still lack adequate access to sanitation and safe drinking water as well as access to basic health services including antenatal care, infant immunizations, and the presence of a skilled attendant at birth [8,9]. Indeed, South Asia’s achievement of the Millennium Development Goals (MDG) has been mixed. Poverty levels were lowered by 54.7%, exceeding the MDG target of 50% and the target for universal primary education enrollment was met, but not secondary school enrollment [10]. South Asia as a region also made notable progress in reducing the maternal mortality rate by 67% between 1990 and 2015; however, the region was unable to meet the targets set for either child mortality or improved sanitation. Similarly, in 2014, only 52% of deliveries were attended by a skilled health professional [10]. While research has demonstrated the importance of water, sanitation, and hygiene (WASH) for maternal and child health (MCH) [11,12], access to improved water and sanitation facilities and use of basic MCH health services have rarely been considered together. Recently, a handful of programs that aimed to increase women’s use of antenatal care services also included water treatment [13] and hygiene education [14,15], pointing towards potential opportunities for MCH and WASH integrated intervention strategies in resource constraint setting.

### Theoretical Background and Study Aims

According to Fundamental Cause Theory derived from Geoffrey Rose’s Cause of Causes, which highlights the role of socioeconomic deprivation as a causal determinant of health [16,17], access to improved water and sanitation facilities, as well as MCH care access and utilization can be considered intervening mechanisms for underlying material deprivation and structural disadvantage of low-socioeconomic status (SES) women and children in South Asia [16,17,18]. Additionally, from a socio-ecological systems perspective, dynamic interrelations exist between individual-level care-seeking behavior and community-level services and infrastructure [19]. Thus, elucidating possible linkages between availability of improved water and sanitation with use of MCH services may lead to refined and thus more effective programs and intervention strategies. Therefore, the aim of this study was to examine associations of household availability of improved drinking water and sanitation, respectively, with reported usage of basic MCH health services, namely antenatal care, presence of a skilled attendant at birth, and infant vaccination coverage based on a large population sample from the most recent Demographic and Health Surveys (DHS) in four South Asian countries.

## 2. Materials and Methods

### 2.1. Study Design and Population

Data for the present study were obtained from the Demographic and Health Surveys (DHS), which collect nationally representative, individual-level data in low- and middle-income countries through a stratified two-stage cluster design [20]. Within each selected household, questionnaires are administered by in-person interviews for all female residents between the ages of 15–49 years and men aged 15–64 years, and data are collected for children under 5 years of age. Verbal informed consent was obtained from all DHS respondents through the reading of a prescribed statement that is written in the questionnaire by the interviewer. More details on the DHS assessments can be found elsewhere [20].

Data from South Asian countries were included in our study if there was at least one DHS survey assessment available after the year 2000. We excluded Afghanistan and the Maldives as their socioeconomic distributions varied greatly from the other countries; thus, we included women’s assessments in Bangladesh, India, Nepal, and Pakistan. As per DHS guidelines on using pooled data, we de-normalized standard weights for each respondent and then re-weighted using UN population division measures on total females aged 15–49 years in the country at the time of survey administration [21,22]. Our current analysis was restricted to women who had a living, singleton birth child, 0–36 months of age at the time of interview.

### 2.2. Water and Sanitation

Households’ types of available water sources and sanitation facilities reported in the DHS were categorized based on the World Health Organization (WHO) and United Nations International Children’s Emergency Fund (UNICEF) definition of “improved” and “unimproved” (Table 1) [23]. We further examined time (recorded as walking time in minutes) to respondent’s water source, re-coded as binary variable (water on premises = 0 min; vs. water not on premises = 1+ min). However, the majority (>90%) of all individuals reported having access to water on premises; thus, we conducted a subgroup analysis restricted to women considered particularly vulnerable, i.e., those in the lowest wealth index group with only unimproved water sources available, to assess whether or not walking time to a water source played a role among this group.

### 2.3. MCH Services

MCH service utilization was defined based on children’s up-to-date immunizations, maternal report of number of antenatal care (ANC) visits and whether a skilled attendant was present at delivery. We considered the WHO’s recommendations for routine immunizations when determining whether a child had up-to-date immunizations. Thus, if the child had received appropriate doses of bacille Calmette–Guerin (BCG), diphtheria, pertussis, and tetanus (DPT), and polio and measles vaccinations based on their given age, he/she was considered to have up-to-date immunizations. We were limited to these four vaccines as the DHS data do not contain sufficient information regarding other childhood vaccinations. Similar to other studies, we defined adequate antenatal care as four or more visits during pregnancy [24,25]. We additionally explored any ANC visits (1 or more; results not shown) and the recent WHO recommendation of 8 or more ANC visits [26]. Women were coded as having a skilled attendant at delivery if they reported a doctor, nurse, midwife, auxiliary midwife, or other healthcare professional were present, while traditional attendants, friends, and/or relatives were considered unskilled (Table 1).

### 2.4. Statistical Analyses

We applied logistic regression models to estimate the odds ratios (OR) and 95% confidence intervals (CI) for improved (vs. unimproved) water sources and sanitation facilities, respectively, and women’s use of health services (as defined in Table 1). Potential confounders were identified a priori, based on previous research examining water and sanitation practices in relation to reproductive and infant health. Our adjusted models included the respondent’s age (grouped in 5-year increments), marital status (not married, married or living with partner), the child’s birth order, the child’s sex, the child’s age in months, urban/rural residency, and log GDP per capita. All models including water sources and time to water sources were also adjusted for type of sanitation facilities and the treatment of water (coded as 1 if respondents boiled water, added bleach or chlorine to water, used a water filter or solar disinfection system; otherwise coded as 0). Similarly, analyses examining associations with sanitation facilities were adjusted for type of water source and whether or not hand-washing facilities were present on site. We further included fixed effects for country and survey year in order to adjust for unobserved time-invariant confounders specific to each country and temporal trends that are shared across countries. Additional adjustments for partner’s age, partner’s educational attainment, and women’s working status did not meaningfully alter estimates of interest and were thus not retained in our models [27]. All analyses were stratified by maternal educational attainment in order to examine possible effect measure modification (“lower educational attainment”, no education or incomplete primary; “higher educational attainment”, complete primary, incomplete secondary, complete secondary, or higher). In sensitivity analyses, we additionally stratified by lower vs. higher wealth index. The DHS wealth index quintile is a measure of household SES and is calculated using information regarding household assets (e.g., television or radio), housing construction, water access, and sanitation facilities [28]. In the DHS, each family is assigned to a wealth index quintile; we grouped those in the bottom two quintiles as “lower wealth index” and those in the top three as “higher wealth index”. Further sensitivity analyses were stratified by urban and rural residency, and we examined tertiles and quartiles of “time to water source”. All analyses were conducted using Stata Statistical Software: Release 14. As the data used for the present study are de-identified and publicly accessible online, the study did not require review from an internal ethics committee.

## 3. Results

Our study sample included data of 145,262 women. Stratifying demographic characteristics of all women by country indicated that mean age, marital status, and distribution of household wealth did not vary considerably between countries (Table 2). Greater proportions of Pakistani women had no education and of women in India and Bangladesh, were residing in rural communities. Additionally, a greater proportion of women with higher education than with lower education had children with up-to-date immunizations, adequate ANC visits, a skilled attendant at delivery, and access to improved sanitation facilities across countries (Table 3).

Combined across countries, low-educated women with improved water sources had greater ORs of having children with up-to-date immunizations (OR: 1.29; 95% CI: 1.17, 1.42) and to have had a skilled attendant at delivery (OR: 1.26; 95% CI: 1.13; 1.37), compared to low-educated women without access to improved water sources. Among women with higher education, there was little indication that availability of improved water source was associated with MCH services usage (Table 4).

Having improved (vs. unimproved) sanitation facilities available was associated with greater ORs of child’s up-to-date immunizations, adequate ANC visits, and the presence of a skilled birth attendant among women in both lower and higher education groups (Table 5). Interestingly, the frequency of women who reported available improved vs. unimproved sanitation facilities did not differ by type of available water sources (Table S1). In both the lower and higher education categories, the majority of women had access to improved water sources (86% and 82%, respectively); however, availability of improved sanitation facilities was about double in the higher education group (59%) compared to the lower education group (29%; Table S2).

Among the vulnerable subgroup of women in the lower wealth index group who did not have an improved water source, not having water on the premises (vs. with water on premises) was associated with an OR of 0.83 (95% CI: 0.71, 0.97) for having had a skilled attendant at birth (Table 6). Sensitivity analyses, stratifying by wealth index instead of educational attainment, resulted in similar odds ratios for models examining associations between type of water source and MCH service usage (Table S3), however, odds ratios related to improved sanitation facility and MCH service usage were smaller (Table S4). Stratifying by urban/rural residency indicated a similar pattern of associations as the high/low education strati. Dividing “time to water” into tertiles or quartiles resulted in lower point estimates, and widened confidence intervals and did not suggest a dose-response association (results not shown).

## 4. Discussion

To our knowledge, our study is the first to examine population-level associations between availability of improved water and sanitation facilities, respectively, and the use of MCH services. Among lower educated women, those with improved water source available had greater ORs for presence of a skilled attendant at delivery and their children having up-to-date immunizations, than those without improved water sources. This may indicate that women with low SES who have no access to improved water may be at particular risk of lacking access to essential MCH services. Interestingly, access to improved sanitation facilities (vs. unimproved facilities) related to greater ORs for having adequate ANC visits, children with up-to-date immunizations, and a skilled birth attendant for both lower and higher educated women. These findings may suggest that increasing access to improved water source among lower socioeconomic groups and to improved sanitation facilitates among all women, may be a strategy that could be linked with access and utilization of MCH services, thereby contributing on multiple levels to maternal and child health in South Asia. 

There are several underlying pathways through which unimproved water and sanitation may relate to lower ORs for utilizing MCH services, with pathogen transmission playing a possible role. According to the WHO, 829,000 deaths from diarrheal disease were related to the quality of WASH in 2016 [29]. Associations between WASH and disease burden are well established [30,31,32]. Maternal impaired health status due to WASH related illness may affect her physical ability to access and use MCH services; this is supported by findings that distance plays a role in likelihood of facility delivery [33] and that good health status in the mother was suggested to be positively associated with antenatal care utilization [34]. Thus, it may be one plausible explanation that women who are ill from contaminated water or non-hygienic sanitation may be less likely to seek care, or attend ANC and vaccination appointments after birth. Caring for ill children or family members may similarly prevent them from seeking or attending adequate services [35]. In our data, improved sanitation was associated with ANC visits among women in both the lower and higher education groups, while improved water only mattered among lower educated women. This finding is in line with a recent study conducted across 41 low- and middle-income countries showing that only unimproved sanitation, and not unimproved water sources, was associated with childhood diarrhea; this study did not stratify by maternal education as we did, and thus could not disentangle possible related differences [11]. Additionally, it is not uncommon for “improved water”, based on current definitions, to contain fecal contamination [36], resulting in misclassification of pathogen exposure; however, this is unlikely to explain differential associations by socioeconomic status. Another explanation may relate to place of residence that enables access to improved WASH facilities and MCH services through infrastructure, and/or co-occurrence of MCH and WASH programs [37]. For example, a typical community setting may entail governmental and other civil society organizations’ programs and may offer a range of programs in support of access to improved WASH and basic MCH services. Moreover, among the women with low SES and without access to improved water sources, those with additional lack of access to a water source on the residential premises had lower OR of having a skilled attendant at birth, when compared to women who had water on the premises. This may indicate a group with very little resources facing multiple constraints in having a skilled attendant at birth, thus pointing out a group for targeted intervention efforts. While there are conceivable linkages between WASH and MCH services, there are few reports addressing potential benefits and challenges of integrated programming. A few, mostly small, intervention studies examined the usage of antenatal service as an entry to other public health interventions, including water treatment and hygiene education [13], or HIV testing and treatment [13,15]. Future operational research is needed to reveal potential benefits and challenges of integrated WASH and MCH programming.

Several limitations need to be considered. The design of our study is cross-sectional limiting interpretation about directionality of the estimated associations. Our study examined access to improved vs. unimproved water sources and, based on available data, cannot differentiate between the use of different water sources for hygiene practices (washing hands, washing dishes etc.) vs. cooking and other consumption. Although we adjusted for “appropriate treatment of water” which did not appreciably change our findings, we have no information on microbial or toxic contamination of the drinking water or other water quality indicators. Indeed, it has been shown that improved water sources, using a similar definition to the one we applied in our analyses [23], can have similar pathogen levels as unimproved water [36]. Thus, pathogen contamination misclassification might potentially weaken associations. Other desirable information we did not have include mothers’ health status or distance to services. While our findings may inform the design and implementation of integrated intervention approaches, additional transdisciplinary and operational research including mixed methods research would be warranted to further understand the possible role of WASH in relation to access and utilization of MCH services, and potential leverage points. Importantly, the COVID-19 pandemic is having substantial impacts, partly in opposite directions, with WASH being scaled up in some areas [38] but not in others, especially not in the most vulnerable constraint resource settings [39,40]; at the same time, research across the globe indicates that access and utilization of MCH services has been reduced related to the pandemic [41]. These developments are crucial to be considered in the current global health agenda.

## 5. Conclusions

The WHO’s “Health in All Policies” approach provides a framework based on the understanding that our greatest health challenges, including health inequities, are highly complex and often linked through social determinants of health, encouraging policy makers to promote health across sectors in public policies to improve maternal and child health equity, targeting the most vulnerable populations. Our findings provide support to the notion that maternal and child health might benefit from approaches across sectors addressing WASH and MCH services [42]. 

## Figures and Tables

**Table 1:** WHO definitions applied to coding of variables.

Variable	Category	Definition
Water ^a^	Improved	Water that came from a pipe, borehole, protected dug well, protected spring, filtration plant, or rainwater
	Unimproved	Water that came from unprotected dug well, unprotected spring, surface water, vendor-provided water, bottled water, or tanker truck water
Sanitation ^b^	Improved	Pour-flush latrine, simple pit latrine, ventilated improved pit latrine, pit latrine with slab, composting toilet, or if toilet flushed to a public sewer or septic system
	Unimproved	Pit latrine without slab, open-pit latrine, bush, field, no toilet facility, bucket toilet, traditional dry vault, dry toilet, or toilet that flushed to “somewhere else”
Birth Attendant ^c^	Skilled	Doctor, nurse, midwife, auxiliary midwife, or other health care professional present
	Unskilled	Traditional attendants, friends and/or relatives
Up-to-date immunizations ^d^	0 to <4 months	BCG (1 dose) Polio (1 doses) DPT (1 dose)
	4 to <6 months	BCG (1 dose) Polio (2 doses) DPT (2 doses)
	6 to ≤12 months	BCG (1 dose) Polio (3 doses) DPT (3 doses)
	13 to ≤36 months	BCG (1 dose) Polio (3 doses) DPT (3 doses) Measles (1 dose)

^a^ The DHS survey asked all women: “What is the main source of drinking water for members of your household?” ^b^ The DHS survey asked all women: “What kind of toilet facility do members of your household usually use?” ^c^ The DHS survey asked all women with children: “Who assisted with the delivery of (NAME)?” ^d^ The DHS survey copied vaccination information from each child’s vaccination card.

**Table 2:** Demographic characteristics of all women (ages 15–49 years) with children, whose last-born child is a singleton birth, alive, and between 0–36 months of age at time of interview.

Demographic Characteristics	Bangladesh	India	Nepal	Pakistan	All
Survey Year	2014	2015–2016	2016	2012–2013	2012–2016
	*N* = 4470 (%)	*N* = 132,667 (%)	*N* = 2687 (%)	*N* = 5438 (%)	*N* = 145,262 (%)
Mean age (SD)	24.6 (0.09)	26.4 (0.01)	25.3 (0.10)	28.5 (0.08)	26.4 (0.01)
Educational Attainment					
No education	600 (13.4)	37,232 (28.1)	767 (28.5)	2944 (54.1)	41,543 (28.6)
Incomplete primary	695 (15.6)	8069 (6.1)	333 (12.4)	303 (5.6)	9400 (6.5)
Complete primary	520 (11.6)	10,276 (7.8)	184 (6.9)	500 (9.2)	11,480 (7.9)
Incomplete secondary	1803 (40.3)	50,583 (38.1)	741 (27.6)	474 (8.7)	53,601 (36.9)
Complete secondary	328 (7.3)	12,232 (9.2)	233 (8.7)	552 (10.2)	13,345 (9.2)
Higher than secondary	524 (11.7)	14,275 (10.8)	429 (16.0)	665 (12.2)	15,893 (10.9)
Marital Status					
Not married	51 (1.1)	1774 (1.3)	12 (0.5)	38 (0.7)	1875 (1.29)
Married or living with partner	4419 (98.9)	130,893 (98.7)	2675 (99.6)	5400 (99.3)	143,387 (98.7)
Residency status					
Urban	1435 (32.1)	31,958 (24.1)	1535 (57.1)	2343 (43.1)	37,271 (25.7)
Rural	3035 (67.9)	100,709 (75.9)	1152 (42.9)	3095 (56.9)	107,991 (74.3)
Household Wealth Index ^a^					
Poorest	933 (20.9)	33,095 (25.0)	698 (26.0)	1186 (21.8)	35,912 (24.7)
Poorer	842 (18.8)	30,815 (23.2)	582 (21.7)	1083 (19.9)	33,322 (22.9)
Middle	865 (19.4)	26,749 (20.2)	554 (20.6)	1042 (19.2)	29,210 (20.1)
Richer	943 (21.1)	22,784 (17.2)	511 (19.0)	1038 (19.1)	25,276 (17.4)
Richest	887 (19.8)	19,224 (14.5)	342 (12.7)	1089 (20.0)	21,542 (14.8)

^a^ The DHS wealth index quintile is a measure of household socioeconomic status (SES) and is calculated using information regarding household assets (e.g., television or radio), housing construction, water access, and sanitation facilities.

**Table 3:** Frequencies of maternal and child health service utilization and of access to improved water and sanitation by country and education.

	Bangladesh		India		Nepal		Pakistan		All	
	*N* = 4470 (%)		*N* = 132,667 (%)		*N* = 2687 (%)		*N* = 5438 (%)		*N* = 145,262 (%)	
	Low ^a^ (%)	High ^b^ (%)	Low ^a^ (%)	High ^b^ (%)	Low ^a^ (%)	High ^b^ (%)	Low ^a^ (%)	High ^b^ (%)	Low ^a^ (%)	High ^b^ (%)
	*N* = 1295	*N* = 3175	*N* = 45,301	*N* = 87,366	*N* = 1100	*N* = 1587	*N* = 3247	*N* = 2191	*N* = 50,943	*N* = 94,319
Up-to-date immunizations	913 (70.5)	2649 (83.4)	22,145 (48.9)	54,947 (62.9)	750 (68.2)	1262 (79.5)	1107 (34.1)	1411 (64.5)	24,915 (48.1)	60,269 (63.9)
ANC visits (4 or more)	222 (17.1)	1165 (36.7)	13,337 (29.4)	49,064 (56.2)	621 (56.5)	1305 (82.2)	712 (21.9)	1392 (63.5)	14,892 (29.23)	52,926 (56.1)
ANC visits (8 or more)	31 (2.4)	246 (7.8)	3180 (7.0)	17,694 (20.3)	40 (3.6)	147 (9.3)	152 (4.7)	517 (23.6)	3403 (6.7)	18,604 (19.7)
Presence of skilled attendant at delivery	288 (22.2)	1625 (51.2)	30,231 (66.7)	75,987 (87.0)	551 (50.1)	1239 (78.1)	1413 (43.5)	1717 (78.4)	32,483 (63.8)	80,568 (85.4)
Improved water sources	1176 (90.8)	2735 (86.1)	37,491 (82.8)	71,264 (81.6)	941 (85.6)	1343 (84.6)	2647 (81.5)	1891 (86.3)	42,255 (83.0)	77,233 (81.9)
Improved sanitation facilities	622 (48.0)	2145 (67.8)	12,400 (27.4)	51,556 (59.0)	706 (64.2)	1332 (83.9)	1975 (60.8)	1901 (86.8)	15,703 (30.8)	56,943 (60.4)

^a^ Low education was defined as none or incomplete primary. ^b^ High education was defined as complete primary or higher.

**Table 4:** Associations between improved (vs. unimproved) water sources and indicators of maternal and child health care service utilization stratified by maternal education.

MCH Service Indicators	All *N* ^a^	Unimproved Water (%)	Improved Water (%)	Crude OR (95% CI)	Adjusted OR (95% CI) ^b^
**Lower education (none or incomplete primary)**					
Up-to-date immunizations	50,943	45.0	51.9	1.31 (1.19, 1.45)	1.29 (1.17, 1.42)
ANC visits (4 or more)	50,943	32.6	30.2	0.89 (0.80, 1.00)	0.90 (0.81, 1.00)
ANC visits (8 or more)	50,943	7.6	7.5	0.99 (0.83, 1.17)	0.99 (0.83, 1.18)
Skilled attendant at delivery	50,943	62.7	66.2	1.16 (1.04, 1.29)	1.26 (1.13, 1.37)
**Higher education (complete primary or higher)**					
Up-to-date immunizations	94,319	64.5	66.7	1.10 (1.02, 1.19)	1.06 (0.99, 1.15)
ANC visits (4 or more)	94,913	62.0	60.0	0.92 (0.85, 1.00)	0.92 (0.85, 1.00)
ANC visits (8 or more)	94,913	25.4	24.7	0.97 (0.88, 1.06)	0.97 (0.88, 1.07)
Skilled attendant at delivery	94,913	88.4	87.9	0.94 (0.84, 1.06)	1.07 (0.94, 1.21)

^a^ Counts obtained using population weights. ^b^ Adjusted for country and year fixed effects, respondent’s age, marital status, child’s birth order, child’s sex, child’s age in months, urban/rural residency, treatment of water, type of sanitation, and log GDP per capita.

**Table 5:** Associations between improved (vs. unimproved) sanitation facilities and indicators of maternal and child health care service utilization, stratified by maternal education.

MCH Service Indicators	All *N* ^a^	Unimproved Sanitation (%)	Improved Sanitation (%)	Crude OR (95% CI)	Adjusted OR(95% CI) ^b^
**Lower education (none or incomplete primary)**					
Up-to-date immunizations	50,943	49.3	55.6	1.29 (1.21, 1.37)	1.27 (1.19, 1.36)
ANC visits (4 or more)	50,943	26.0	40.6	1.95 (1.83, 2.09)	1.75 (1.62, 1.88)
ANC visits (8 or more)	50,943	6.1	10.9	1.89 (1.68, 2.13)	1.67 (1.46, 1.92)
Skilled attendant at delivery	50,943	64.6	68.8	1.21 (1.13, 1.30)	1.37 (1.27, 1.47)
**Higher education (complete primary or higher)**					
Up-to-date immunizations	94,319	61.5	69.1	1.40 (1.33, 1.47)	1.31 (1.24, 1.38)
ANC visits (4 or more)	94,319	48.5	66.5	2.14 (2.05, 2.24)	1.71 (1.62, 1.80)
ANC visits (8 or more)	94,319	16.8	29.1	2.04 (1.91,2.17)	1.64 (1.52, 1.76)
Skilled attendant at delivery	94,319	83.7	90.3	1.85 (1.73, 1.97)	1.67 (1.55, 1.79)

^a^ Counts obtained using population weights. ^b^ Adjusted for country and year fixed effects, respondent’s age, marital status, child’s birth order, child’s sex, child’s age in months, urban/rural residency, type of water source, presence of hand washing facilities on site, and log GDP per capita.

**Table 6:** Associations between walking time (in minutes) to water source and indicators of maternal and child health care services utilization among vulnerable women ^a^.

		Up-to-Date Immunizations		ANC Visits (4 or More)		Skilled Attendant at Birth	
Time to water source	*N* = 9836 (%)	Crude OR	Adjusted OR (95% CI) ^b^	Crude OR	Adjusted OR (95% CI) ^b^	Crude OR	Adjusted OR (95% CI) ^b^
Water on premises (0 min)	27.5	ref	ref	ref	ref	ref	ref
Water not on premises (>0 min)	72.4	0.88 (0.77, 1.01)	0.96 (0.84, 1.10)	0.96 (0.82, 1.13)	1.12 (0.95, 1.31)	0.73 (0.62, 0.85)	0.83 (0.71, 0.97)

Ref = reference category. ^a^ Subgroup of women without access to improved water sources and in a low wealth index (poorest, poorer). ^b^ Adjusted for country and year fixed effects, respondent’s age, educational attainment, marital status, urban/rural residency, child’s birth order, child’s sex, child’s age in months, appropriate treatment of water, type of sanitation, and log GDP per capita.

## Data Availability

All Demographic and Health Survey data used in the present study are available free for download at https://dhsprogram.com (accessed on 1 June 2018).

## Supplementary Materials

The following are available online at https://www.mdpi.com/article/10.3390/ijerph18147667/s1. Table S1: Cross tabulation of women who have access to improved and unimproved water vs. improved and unimproved sanitation. Table S2: Cross tabulation and Spearman correlation of women who have access to improved and unimproved water and sanitation stratified by education. Table S3: Associations between improved (vs. unimproved) water sources and indicators of maternal and child health care services by DHS wealth index. Table S4: Association between improved (vs. unimproved) sanitation facilities and indicators of maternal and child health care services by DHS wealth index.
