# Supplementary material for: Improved Water, Sanitation and Utilization of Maternal and Child Health Services in South Asia—An Analysis of Demographic Health Surveys
Source: Int J Environ Res Public Health. 2021 Jul 19;18(14):7667. doi: 10.3390/ijerph18147667 (PMC8303440; doi:10.3390/ijerph18147667)
Supplement: Supplementary file 1 [file ijerph-18-07667-s001.zip › Supplemental Table 1.pdf]

Table S1. Cross tabulation of women who have access to improved and unimproved water vs. improved and unimproved sanitation.

	**Unimproved sanitation**	**Improved sanitation**	**Missing**
	(%)	(%)	(%)
**Unimproved water**	53.0	46.9	0.11
**Improved water**	46.4	53.4	0.18
**Missing**	0.8	1.0	98.2
