# Supplementary material for: Improved Water, Sanitation and Utilization of Maternal and Child Health Services in South Asia—An Analysis of Demographic Health Surveys
Source: Int J Environ Res Public Health. 2021 Jul 19;18(14):7667. doi: 10.3390/ijerph18147667 (PMC8303440; doi:10.3390/ijerph18147667)
Supplement: Supplementary file 1 [file ijerph-18-07667-s001.zip › Supplemental Table 2.pdf]

Table S2. Cross tabulation and Spearman correlation of women who have access to improved and unimproved water and sanitation stratified by education.

	Lower Education	Higher Education	Correlation
	**(%)**	**(%)**	
**Improved Water**	85.6	82.4	
**Unimproved Water**	9.1	9.0	
**Missing**	5.3	8.6	0.0306
	**(%)**	**(%)**	
**Improved Sanitation**	28.8	59.2	
**Unimproved Sanitation**	66.0	32.1	
**Missing**	5.3	8.7	0.2886
