# Supplementary material for: Improved Water, Sanitation and Utilization of Maternal and Child Health Services in South Asia—An Analysis of Demographic Health Surveys
Source: Int J Environ Res Public Health. 2021 Jul 19;18(14):7667. doi: 10.3390/ijerph18147667 (PMC8303440; doi:10.3390/ijerph18147667)
Supplement: Supplementary file 1 [file ijerph-18-07667-s001.zip › Supplemental Table 3.pdf]

Table S3. Associations between improved (vs. unimproved) water sources and indicators of maternal and child health care services by DHS wealth index.

Reproductive and infant health outcomes	All N	With MCH service and unimproved water (%)	With MCH service and improved water (%)	Crude OR (95% CI)	Adjusted OR (95% CI)[a]
**Lower wealth index (poorest, poorer)**					
Up-to-date immunizations	69,234	48.3	55.4	1.33 (1.24, 1.43)	1.30 (1.20, 1.40)
Adequate ANC visits (4 or more)	69,234	34.7	33.3	0.94 (0.86, 1.02)	0.95 (0.88, 1.04)
Adequate ANC visits (8 or more)	69,234	8.1	8.8	1.10 (0.94, 1.29)	1.06 (0.91, 1.23)
Skilled attendant at delivery	69,234	66.1	70.0	1.20 (1.09, 1.31)	1.28 (1.19, 1.39)
**Higher wealth index (middle, richer, richest)**					
Up-to-date immunizations	76,028	66.4	66.6	1.01 (0.92, 1.11)	0.99 (0.90, 1.09)
Adequate ANC visits (4 or more)	76,028	67.3	63.1	0.84 (0.76, 0.92)	0.88 (0.80, 0.97)
Adequate ANC visits (8 or more)	76,028	29.4	26.9	0.89 (0.80, 0.99)	0.92 (0.83, 1.03)
Skilled attendant at delivery	76,028	91.7	89.0	0.69 (0.57, 0.82)	0.88 (0.73, 1.06)

[a]Adjusted for country and year fixed effects, respondent's age, educational attainment, marital status, child's birth order, child's sex, child's age in months, urban/rural residency, appropriate treatment of water, improved sanitation and log GDP per capita.
