# Supplementary material for: Improved Water, Sanitation and Utilization of Maternal and Child Health Services in South Asia—An Analysis of Demographic Health Surveys
Source: Int J Environ Res Public Health. 2021 Jul 19;18(14):7667. doi: 10.3390/ijerph18147667 (PMC8303440; doi:10.3390/ijerph18147667)
Supplement: Supplementary file 1 [file ijerph-18-07667-s001.zip › Supplemental Table 4.pdf]

**Table S4.** Association between improved (vs. unimproved) sanitation facilities and indicators of maternal and child health care services by DHS wealth index.

| Reproductive and infant health outcomes | All N | With MCH service and unimproved Sanitation N (%) | With MCH service and improved Sanitation N (%) | Crude OR (95% CI) | Adjusted OR (95% CI)[a] |
|---|---|---|---|---|---|
| **Lower wealth index (poorest, poorer)** | | | | | |
| Up-to-date immunizations | 69,234 | 52.9 | 61.4 | 1.42 (1.33, 1.50) | 1.26 (1.18, 1.34) |
| Adequate ANC visits (4 or more) | 69,234 | 30.5 | 44.7 | 1.85 (1.73, 1.98) | 1.59 (1.48, 1.71) |
| Adequate ANC visits (8 or more) | 69,234 | 7.8 | 12.2 | 1.64 (1.46, 1.85) | 1.42 (1.25, 1.61) |
| Skilled attendant at delivery | 69,234 | 69.7 | 69.0 | 0.97 (0.91, 1.04) | 1.05 (0.98, 1.13) |
| **Higher wealth index (middle, richer, richest)** | | | | | |
| Up-to-date immunizations | 76,028 | 62.8 | 67.5 | 1.23 (1.16, 1.31) | 1.14 (1.07, 1.22) |
| Adequate ANC visits (4 or more) | 76,028 | 57.7 | 64.9 | 1.37 (1.29, 1.46) | 1.11 (1.04, 1.18) |
| Adequate ANC visits (8 or more) | 76,028 | 22.6 | 28.3 | 1.36 (1.26, 1.47) | 1.08 (0.99, 1.17) |
| Skilled attendant at delivery | 76,028 | 87.3 | 89.7 | 1.25 (1.15, 1.37) | 1.09 (0.99, 1.20) |

[a]Adjusted for country and year fixed effects, respondent's age, educational attainment, marital status, child's birth order, child's sex, child's age in months, urban/rural residency, presence of improved water sources, presence of hand washing facilities on site and log GDP per capita.
